# The Liver, Viewed as a Sugar-Producer

**Published:** 1855-05

**Authors:** William Howell


### ART. VI.—*The Liver viewed as a Sugar Producer.*

In writing a thesis on the production of sugar by the liver, I was unable to obtain the results of some observations by Valentin, relative to the condition in respect of sugar, of the organs of the hybernating animals, during hybernation. Having since obtained them, I beg leave herewith to present them.

In the first observation made upon a marmot, which had begun hybernation but four days before, but which had eaten nothing for a week, the stomach being quite void of food, and containing only the grayish-white neutral substance, normally its occupant during hybernation, the liver was found to afford a large quantity of sugar. With a view to a more thorough examination, a second marmot (male) was taken, which, having eaten nothing for some days before hybernation commenced, lay asleep for twenty-five days. It then woke up, but, falling asleep again on the following day, it remained for eleven days in a condition of perfect winter-sleep. It then, for the second time, awoke, passed urine and fæces for the first time since hybernation commenced, and fell asleep again on the next day. Three days after it was asphyxiated by being placed *in vacuo*. At the commencement of hybernation its weight was $2\frac{1}{8}$ pounds; at death it had diminished nearly three ounces, about $\frac{1}{12}$ of its weight. A solution of the liver, treated by the test of fermentation, by that of copper, by the potash-test, and finally by polarization, distinctly evinced the presence of sugar. The economy of which it was a part had been unsupplied with food for six weeks. From a quantitative analysis it appeared that the organ contained 2.87 per cent. by weight of sugar. Six decoctions were necessary before the liver was completely exhausted of its sugar. But not only in the parenchyma of the liver, but in the blood which flowed from an incision into it, and which manifested almost total absence of coagulability; in the bile and in the normal contents of the stomach, were found manifest evidences of sugar. In the blood the per centage of sugar was 82, by weight. A filtered decoction of the diaphragm of the right supra-renal capsule, and of the substance of the heart, all yielding faint traces of sugar, though in the last two organs it was very weak indeed. Its presence could not be demonstrated in any other organ.

In remarkable contrast with this extensive diffusion of sugar, is the case of a young hedgehog which was also examined. In this animal hybernation was not at all complete. Rapidity of respiration was decreased only about one-half, deep respiration followed any disturbance of his quiet, he woke up about every week, and died after two months, evidently from starvation. No trace of sugar was obtained from any organ.

When the general conditions which obtain, in hybernation, are taken into account, the facts above stated bear weighty testimony to the truth of the theory of M. Bernard. During the winter sleep of the marmot, the contractions of the heart fall from 150 beats in the minute to 15, while the respirations instead of numbering 500 in the hour, as in the summer, fall to 14. The temperature of the body maintains a pretty uniform ratio with that of the surrounding air, being generally but two or three degrees above it. At 30° F. the temperature of the body is 35°, but as the temperature of the air sinks below this point the cold appears to act as a stimulus, and the animal heat is elevated, though only temporarily and antecedent to a fatal depression of it. This elevation of temperature consequent upon the stimulus of cold, or other external irritation, is a constant fact, and as being consequent upon the processes of nutrition and waste the *capability* of this elevation, taken in connection with the results of the experiments detailed above, is a beautiful extension of M. Bernard's ideas. For from what is generally known as to the chemical capabilities of sugar as a supporter of heat, when, in a case like that of the Hibernants, we find the evolution of animal heat so far prolonged, during the total deprivation of ingesta of all kinds; and further, when we find the sugar in so much greater profusion and over so much greater extent, than it can be found when the functions of the economy (respiration and circulation in particular) are being performed to their full extent, we can arrive at no other conclusion than that this excess of sugar has been elaborated by the liver, the labors of whose office have been increased in consequence of the general suspension or depression of the functions of the other viscera. And that this sugar, although in such excess, is intended for the purpose of the maintenance of the animal heat, to a low degree, for a long space of time.

W. HOWELL, M. D.

